# The road map for ISO 15189-laboratory accreditation: The Experience of Manhiça Health Research Centre (CISM) laboratory, in southern Mozambique

**DOI:** 10.4102/ajlm.v15i1.3153

**Published:** 2026-05-22

**Authors:** Delfino C. Vubil, Anelsio Cossa, Ergel Salvador, Miguel Bene, Geraldo Manhiça, Chenjerai Jairoce, Edson Mambuque, Lázaro Quimice, Eusébio V. Macete, Francisco Saute, Inacio Mandomando

**Affiliations:** 1 Manhiça Health Research Centre (CISM), Maputo, Mozambique; 2 National Institute of Health (INS), Maputo, Mozambique; 3 ISGlobal, Barcelona, Spain

**Keywords:** laboratory accreditation, quality management systems, ISO 15189, CISM, Mozambique

## Abstract

**Background:**

Accreditation is an effective way to recognise the quality and competence of a clinical laboratory. Here we describe the steps towards ISO 15189:2012 accreditation of the Manhiça Health Research Centre laboratory, in Mozambique.

**Intervention:**

The accreditation process started in 2012 with a pre-assessment through the Stepwise Laboratory Improvement Process Towards Accreditation, followed by application to the Portuguese Accreditation Institute in 2014, which conducted two audits in 2018 and 2019. Most findings from the accreditation audits were related to personnel management, equipment and reagents. In 2020, the laboratory obtained the accreditation for ISO 15189:2012 and competence for identification and quantification of *Plasmodium* species by microscopy; full blood count; determination of creatinine, aspartate aminotransferase, alanine aminotransferase, alkaline phosphatase, total bilirubin, gamma glutamyl transferase, urea; CD4 count; identification and quantification of interferon-γ; detection and quantification of *P. falciparum* by real-time polymerase chain reaction; rotavirus genotyping; detection of rotavirus by enzyme-linked immunosorbent assay; and bacterial culture, identification and antimicrobial susceptibility testing.

**Lessons learnt:**

Laboratory accreditation is a complex process, which is only possible with the involvement of the whole organisation. Continuous improvement is essential to achieve accreditation.

**Recommendations:**

In preparation for ISO 15189 accreditation, a medical laboratory should focus on building a robust quality management system to ensure the competence of the whole laboratory.

**What this study adds:**

This study demonstrates the importance of strengthening quality management systems towards accreditation. To our best knowledge, this is the first report of ISO 15189 accreditation in a private non-profit research organisation in Mozambique.

## Background

### Introduction

Reliable clinical laboratory services are essential for a functional health system, as they play a key role in supporting diagnosis, treatment and overall patient management.^1^ However, there is a poor and limited laboratory capacity in most low- and middle-income-settings, including sub-Saharan African (SSA) countries like Mozambique.^2,3^ One way to accomplish these quality standards is to get accreditation through the implementation of quality management systems (QMSs).

Accreditation is a procedure by which an independent, authoritative body gives formal recognition that an organisation or person is competent to carry out specific tasks,^4,5^ whereas a QMS provides the integration of organisation structure, procedures, processes and resources needed to fulfil a quality policy and, therefore, to meet the needs and requirements of users.^6^ The key elements of a laboratory quality assurance programme include providing a functional and safe laboratory environment, trained and competent personnel, maintaining equipment, adequate supplies and reagents, testing appropriate specimens, internal monitoring of quality, accurate reporting, and external quality assessments (EQAs).^7^ These components are necessary to provide accurate and precise laboratory results for patient care, prevention, disease surveillance, and outbreak investigation.^8^

The development and maintenance of high-quality laboratory services require financial and managerial commitment to provide qualified staff, training, equipment, consumables, reagents, and physical facilities. Furthermore, periodic quality assessment of performance is essential to ensure the reliability of findings, and it is an important component of laboratory accreditation.^9^ Failure to meet minimum safety standards may put laboratory workers, patients, and the community at risk.^10^ Training programmes are needed to enable laboratory managers to use available resources efficiently for planning, implementation, and evaluation of service delivery to meet the expectations of patients and clinicians, and public health needs.^2^

Evidence from several high- and low-income settings^3,11,12,13^ demonstrates that the implementation of QMSs and accreditation leads to a measurable improvement in the quality of services and increased patient safety owing to a reduction in laboratory errors. The major gain of accreditation of medical testing laboratories is that results are accepted internationally and the quality of the laboratory is always maintained, leading to customer satisfaction and confidence.^14^ Despite the resource constraints, there is a growing interest in leveraging quality systems in medical laboratories in most low- and middle-income countries (LMICs). A study published in 2023,^15^ which assessed the performance of 7 years of implementation of QMS in medical laboratories in SSA (2013–2020), demonstrated that 668 laboratories had achieved accreditation by 2020, corresponding to an increase of 75% from the number reported in 2013. Most of the accredited laboratories were from South Africa (*n* = 396), followed by Kenya (*n* = 106), which are two countries with national accreditation bodies.^15^ In Mozambique, the majority of accredited laboratories belong to the public sector, with the National Tuberculosis Reference Laboratory (NTRL), being the first medical laboratory to obtain ISO 15189 accreditation in 2015.^16^ Here we describe the steps undertaken by the Manhiça Health Research Centre (CISM) laboratory to comply with ISO 15189:2012 accreditation, in the private sector. The laboratory was certified to ISO 9001:2008 from 2009 to 2015 for QMS through the South African Bureau of Standards (SABS) with the reference number LS4357.

## Description of the Intervention

### Material and methods

#### Design and setting

This is a descriptive and retrospective analysis regarding the steps and challenges for accreditation of the CISM laboratory. Manhiça Health Research Centre is a biomedical research institution, created in 1996 as the result of a cooperative agreement between the governments of Mozambique and Spain (www.cismmanhica.org). The centre is based in Manhiça District, in a rural area of southern Mozambique, 80 km from Maputo, the capital city. Manhiça Health Research Centre conducts research in health priority areas such as malaria, tuberculosis, HIV, diarrhoea, respiratory infections, and neglected tropical diseases, among others. From 1998 until 2024, CISM implemented, around the clock, a morbidity surveillance system in a collaboration with the Manhiça District Hospital, which is the referral health facility for the whole Manhiça District. Through the morbidity surveillance system, the demographic data, signs, symptoms, and diagnoses of all outpatients and inpatients under the age of 15 were collected routinely. The morbidity surveillance system was linked to the health and demographic surveillance system (HDSS) until 2024, from which all vital events were collected regularly from the population living in the Manhiça District.^17^ The data management department is responsible for the storage and management of all generated information, including laboratory databases.

#### The Manhiça Health Research Centre laboratory

The CISM laboratory was established to provide technical and scientific support to research studies while contributing to patient care at the hospital in collaboration with the Manhiça District Hospital. The laboratory is organised into the following areas: blood parasitology, haematology and biochemistry, microbiology, tuberculosis (TB), immunology, molecular biology, entomology, biorepository, and the quality management unit. The blood parasitology laboratory performs malaria diagnosis by optical microscopy (identification and quantification). The microbiology laboratory specialises in culture, isolation, and identification of bacterial pathogens and antimicrobial susceptibility testing. The TB unit is equipped with a level III biosafety laboratory, which also supports the Mozambique national health system, providing TB diagnostics capacity for the whole community of Manhiça District. The immunology laboratory has the capacity for cell isolation and serology. The molecular biology laboratory is equipped with the entire infrastructure needed to perform nucleic acid-based detection methodologies from conventional polymerase chain reaction (PCR) to real-time (q) PCR, including DNA sequencing. The entomology unit includes an insectary that supports malaria vector studies. The biorepository guarantees the long-term storage of biological specimens in ultra-freezers and cryopreservation in liquid nitrogen (LN_2_). The quality management unit is responsible for the implementation and maintenance of the quality system for the whole laboratory. Furthermore, the laboratory has operated under the laboratory information and management system (LIMS)^18^ since 2010, covering the sample reception, processing, results entry, patient reports, and sample storage.

#### The accreditation path

**Transition from ISO 9001:2008 to ISO 15189:2012:** Since its foundation, the CISM laboratory has been implementing a QMS. The laboratory was ISO 9001:2008 certified in 2009 and maintained the certification until 2015. However, this certification does not comply totally with the specific requirements for quality and competence for medical laboratories.

The accreditation process started in 2012 with a pre-assessment through Stepwise Laboratory Improvement Process Towards Accreditation (SLIPTA), conducted by World Health Organization (WHO) consultants. The purpose of the SLIPTA checklist is to evaluate and verify the establishment, implementation, and improvement of the QMS in medical and public health laboratories. The scored checklist allows for the rating of a laboratory’s quality improvement status by using a zero to five-star scale.^8,19^ Laboratories awarded five stars are encouraged strongly to enrol in an established ISO 15189 accreditation scheme.^8^

The improvement process of our laboratory consisted of updating the quality manual, and procedures for the following quality elements: organisation, personnel, equipment, purchases and inventory, process control, information management, documents and records, occurrence management, quality control assessment, process improvement, customer service, laboratory facilities, and safety.^5^ Quality indicators were established and monitored daily, weekly, monthly, or annually, as needed, to assess the effect of the quality of laboratory services. Specimen processing, turnaround times, and rejections for all laboratory tests were monitored using standard questionnaires. We also established a plan for the maintenance of equipment and stock management to ensure the continuity of the work. The risk assessment was implemented covering all laboratory-processing phases: pre-analytic, analytic, and post-analytic. External quality assessment for all tests of the accreditation scope was implemented to assess the laboratory’s performance. The laboratory established a customer satisfaction assessment, where researchers and medical staff give their opinion about the laboratory performance once a year, responding independently to a standard questionnaire. We also implemented an internal audit programme in accordance with ISO 15189:2012 accreditation scope. All quality indicators were collected via a checklist and reviewed monthly by the quality manager.

#### Application process

The application process started in December 2014 through the Portuguese Accreditation Institute (IPAC) (www.ipac.pt). IPAC acts as technical regulator for testing, calibration and clinical laboratories, inspections and certification bodies. The application consisted of the completed application form, proof of existence of the legal entity, submission of a quality manual including procedures, and proof of payment for instruction regarding the process. A unique identification number was issued followed by a preliminary analysis to confirm reception and conformity. This step was followed by the designation of the audit team comprising a lead and two technical experts.

#### Audit process

The first audit was conducted from 01 to 03 October 2018. Through this process, the QMS and technical competence were assessed for blood parasitology, haematology, biochemistry, immunology, molecular biology, bacteriology, and tuberculosis laboratories. After the audit report, we implemented an action plan to resolve each finding. The corrective action plan was submitted to IPAC within 30 days from reception of the audit report. A follow-up assessment was conducted from 07 to 08 September 2019.

Audit findings non-conformities (NC) were classified as minor or major, according to IPAC regulations.^20^ Major findings are defined as the absence or systematic failure in implementing the accreditation requisites with significant implications for the reliability of the results, or for independence or impartiality, or for compliance with the accreditation obligations; while a minor finding refers to a single failure of an accreditation requirement that does not affect significantly the reliability of the results for the activity carried out, or for confidence in the independence or impartiality. This is usually a documentation failure (e.g. correct practice but not documented) or a single and non-serious procedural failure (incorrect practice, but without significant implications).^20^

#### Ethical considerations

Ethical clearance to conduct this study was obtained from the Institutional Bioethics committee. The ethical clearance number is CIBS-CISM/041/2025.

## Lessons learnt

The first step before accreditation is to build an enthusiastic team with education regarding QMS. The involvement of the top management of the organisation is crucial, not only for providing financial support but also for motivating personnel.

The duration of the accreditation process depends on the scope of the accreditation, the complexity of testing operations, the level of laboratory preparation, and the time taken to resolve the audit findings.

### Results

#### Quality improvement programme

The overall duration of the transition programme from ISO 9001:2008 to ISO 15189:2012 was about 8 years (2012 to 2020). In the SLIPTA pre-accreditation audit, the laboratory scored four stars out of five, and recommendations were: to update the quality system to comply with ISO 15189:2012; to train the staff in the reference standard; and to identify clearly the scope of accreditation. The summary of some performance quality indicators for the last 3 years of the accreditation period (2018–2020) are presented below.

**FIGURE 1:**
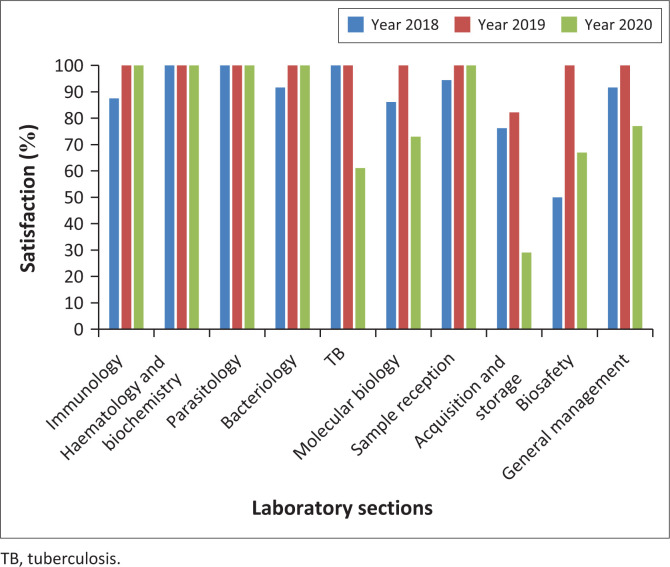
Client satisfaction regarding the laboratory services from January 2018 to December 2020, Manhiça, Mozambique.

In 2019, all the laboratory personnel were trained on the ISO 15189:2012 by RELACRE – Associação de Laboratórios Acreditados de Portugal (https://www.relacre.pt/pt/home). The customer satisfaction survey showed that our clients were satisfied with most of the laboratory services (Figure 1). The degree of satisfaction ranged from 29% to 100%, with the lowest score reported in the section of acquisitions and storage in 2020. Haematology, biochemistry and parasitology scored 100% in the 3 years of evaluation. Other services that scored highest in any of the evaluation periods include immunology, bacteriology and sample reception (2019 and 2020), TB (2018 and 2019), and molecular biology, biosafety and general management in 2019. The specimen rejection was below the preset cut-off (5%), and more than 95% of samples were processed in time.

Our EQA programme demonstrated, in general, a good performance for the majority of methods in the scope of accreditation, over the analysed period (Table 1). The highest performance was observed for the immunology laboratory (QuantiFERON-TB), haematology (full blood count), with 100% achievement over the analysis period. Other laboratories with 100% scores include molecular biology (detection of *Plasmodium falciparum* in 2018 and 2019, and detection and genotyping of rotavirus in 2018), biochemistry gamma glutamyl transferase (GGT) in 2019 and 2020, and creatinine in 2019, and parasitology (identification of *P. falciparum* by microscopy) in 2019.

**TABLE 1:** Performance of external quality assessment from January 2018 to December 2020, Manhiça, Mozambique.

Lab section	Methods/Techniques	Score		
		2018 (%)	2019 (%)	2020 (%)
Parasitology	Identification of *P. falciparum*	80.0	100.0	83.3
Immunology	QuantiFERON-TB	100.0	100.0	100.0
	CD4 count	100.0	100.0	44.0
Tuberculosis (TB)	TB Smear, culture and identification	93.3	96.5	97.3
Bacteriology	Culture, Gram staining, identification and antimicrobial susceptibility testing (AST)	74.0	67.0	90.9
Molecular biology	Detection of *P. falciparum*	100.0	100.0	Not done
	Detection and genotyping of rotavirus	100.0	Not done	Not done
Haematology	Full blood count	100.0	100.0	100.0
Biochemistry	ALT (alanine aminotransferase)	83.0	79.1	50.0
	ALKP (alkaline phosphatase)	91.5	95.8	85.0
	GGT (gamma glutamyl transferase)	91.5	100.0	100.0
	AST (aspartate aminotransferase)	100.0	66.7	70.0
	TBIL (total bilirubin)	83.0	83.3	85.0
	Creatinine	87.5	100.0	75.0
	Urea	66.6	95.8	95.0
	LDH (lactate dehydrogenase)	37.5	Not done	Not done

#### IPAC audit findings

**FIGURE 2:**
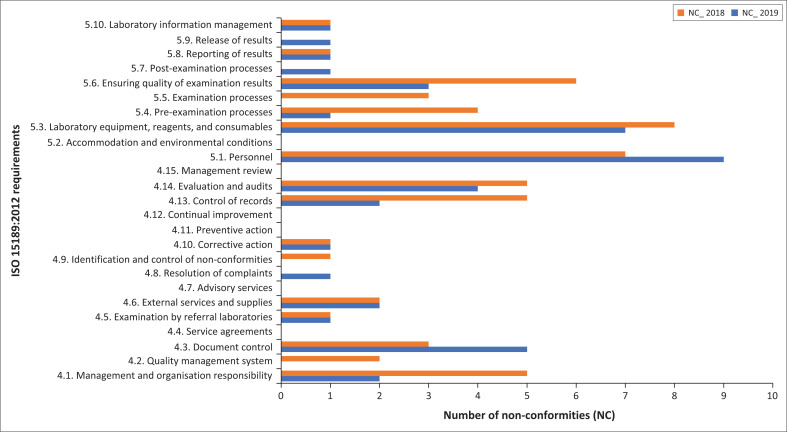
Absolute number of non-conformities per ISO 15189:2012 requirements for the two external audit assessments (01 October–03 October 2018 and 07 September–08 September 2019, Manhiça, Mozambique).

The results from the two onsite audits are presented in Figure 2. Fifty-five minor findings were identified in the first audit, and 42 minor findings in the second audit visit. Most of the findings in both audits were related to personnel management (seven findings in 2018 and nine findings in 2019), equipment and reagents (eight findings in 2018, and seven findings in 2019). All NC were resolved within the required period, as per IPAC regulations.

#### Technical competence

After the successful resolution of all NC, the laboratory was awarded the accreditation certificate on 01 October 2020, and it was deemed competent for the identification and quantification of *Plasmodium* species by microscopy; full blood count; biochemistry analysis (Creatinine, aspartate aminotransferase [AST], alanine aminotransferase [ALT], alkaline phosphatase [ALKP], total bilirubin [TBIL], gamma glutamyl transferase [GGT], Urea); CD4 count; identification and quantification of Interferon-γ; detection and quantification of *P. falciparum* by real-time PCR; rotavirus genotyping by conventional RT-PCR; detection of rotavirus by enzyme-linked immunosorbent assay (ELISA); and bacterial culture, identification and antimicrobial susceptibility testing (Table 2).

**TABLE 2:** Summary of accredited tests/methods by laboratory section as of 01 October 2020, Manhiça, Mozambique.

Laboratory	Sample type	Examination or method
Blood parasitology	Human blood smear slide	Identification, differentiation and quantification of *Plasmodium spp*. by microscopy
Haematology	Human whole blood	Whole blood count
Biochemistry	Human serum	AST (aspartate aminotransferase), ALKP (alkaline phosphatase), ALT (alanine aminotransferase), Creatinine, GGT (gamma glutamyl transferase), TBIL (total bilirubin), Urea
Molecular biology	Human faeces	Detection of rotavirus by enzyme immunoassay (ELISA)
		Rotavirus genotyping by conventional RT-PCR
	Dried human blood on filter paper	Detection and quantification of *P. falciparum* by qPCR
Tuberculosis (TB)	Biological liquids (gastric aspirate, cerebrospinal fluid [CSF], pleural fluid, peritoneal fluid), exudates and human sputum	Acid-fast bacilli (AFB) identification by smear slide staining using Ziehl-Neelsen
		Identification of *Mycobacterium spp*. by culture
		Antimicrobial susceptibility testing (AST)
Immunology	Human blood	Detection and quantification of Interferon-γ by enzyme-linked immunosorbent assay (QuantiFERON-TB)
		Absolute and percentage quantification of CD4 T cells
Bacteriology	Biological liquids (gastric aspirate, cerebrospinal fluid [CSF], pleural fluid, peritoneal fluid), exudates, human sputum, blood, urine and faeces	Gram staining
		Detection of facultative aero-anaerobic bacteria by culture
		Identification of Gram-negative and Gram-positive bacteria
		Antimicrobial susceptibility testing (AST)
	Biological liquids (gastric aspirate, cerebrospinal fluid [CSF], pleural fluid, peritoneal fluid), exudates and human sputum	Capsular antigen detection for *Haemophilus influenzae*; *Streptococcus pneumoniae*; *Neisseria meningitidis*; Group B *Streptococcus* by latex agglutination

qPCR, real-time polymerase chain reaction.

#### Accreditation costs

Most of the accreditation costs are inherent to the implementation of the QMS in compliance with ISO15189, which includes personnel, equipment, reagents and consumables. Besides this, there are the costs of accreditation itself, including fees, and travel logistics for the auditors during site assessments. Table 3 summarises some of the incurred costs for accreditation of CISM laboratory. Most of these costs were related to equipment maintenance and calibration.

**TABLE 3:** Approximate cost for accreditation of CISM laboratory up to December 2020, Manhiça, Mozambique.

Item	Approximate cost (USD)
Training on ISO 15189:2012	5739.20
Annual external quality assessment (EQA)	16 877.00
Annual equipment maintenance and calibration	258 878.00
Application fee to the accreditation board (IPAC)	968.56
Annual audit fees	6286.34
Travel and accommodation for the audit team (each visit)	4909.02

**Total**	**293 658.12**

## Recommendations

In preparation for ISO 15189 accreditation, a medical laboratory should start by understanding the requirements of the standard. The key steps include performing a gap analysis of existing processes against the ISO 15189 requirements and the implementation of procedures to ensure competence.

### Discussion

With the expansion and growth of research activities in the SSA countries, many laboratories have been established; however, only a few of them have a qualification or certification by an external entity. Clinical trials are now demanding that laboratories have a reasonable quality control mechanism through certification or accreditation.

Although there is no preset timeline to complete accreditation, it is estimated usually to take 6 months to 1 year to prepare for the accreditation assessment.^21^ In general, the duration of the accreditation process depends on the scope of the accreditation, the complexity of testing operations, the level of preparation, and the time taken to resolve all findings derived from any audit assessment.^21^ Our laboratory took 2 years to prepare before submission of the application to IPAC in 2014. After that, the laboratory spent almost 4 years responding to the queries from the accreditation board before the appointment of the first accreditation assessment visit, which took place in October 2018. Then, the second follow-up audit in September 2019 culminated in the award of the accreditation in October 2020. This long period before the first audit assessment is explained in part by the limited preparation of the laboratory, as well as by the need to adapt the whole QMS to comply with ISO 15189 requirements. This gap was identified in the SLIPTA pre-accreditation audit conducted in 2012, including the need to train the staff in the reference standard and to identify clearly the scope of accreditation. In fact, the lack of training in ISO 15189:2012 was also highlighted in the first accreditation audit conducted by IPAC in 2018; therefore, to solve this finding, all laboratory staff were trained in 2019 before the second audit assessment. All these aspects reinforce the importance of laboratory preparation for the duration of the accreditation process (the more the laboratory is prepared; the shorter the duration).

Through this journey, we also learnt that the first step before accreditation is building an enthusiastic team through education about QMS. Therefore, the laboratory personnel had to work together towards the implementation of the quality improvement plan, which included the selection of methods, definition and structuring of documents, preparation of a quality manual, procedures and regular competence assessment. All staff had to be trained on ISO 15189:2012 by RELACRE. Indeed, continuing education is essential for personnel who participate in management and technical processes and the effectiveness of the programme should be reviewed periodically.^4^ The involvement of the top management of the organisation was also critical for this achievement, not only for providing financial support but also through active participation in the whole process, including during the external audits. Actually, the role of institutional support for laboratory accreditation has been demonstrated in other African countries,^22,23^ including in the accreditation of the National Tuberculosis Reference Laboratory in Mozambique.^16^

Both external and internal audits were crucial in determining the areas that needed improvement and for monitoring the action plans. As shown in Figure 2, most of the IPAC audit findings were associated with personnel management, equipment, and reagents, which were consistent with our internal audit findings. These findings are among the most common NC from laboratory assessments.^24^ In general, the number of findings decreased in the second audit conducted in 2019, compared to the first assessment in 2018. The reduction of NC demonstrates the effectiveness of the action plan implemented to improve the laboratory quality system. This observation is sustained in part by the overall client satisfaction (Figure 1) and the good performance of the EQA programme (Table 1). Indeed, EQA performance was one of the criteria for method selection, and it is a requirement for ISO 15189 accreditation.^25^

Accreditation is a complex process that has many challenges, particularly in LMICs like Mozambique. One important aspect to consider is the cost of the accreditation process itself, besides the costs related to the implementation of the QMS (Table 3). These costs include the accreditation fees, which may vary according to the accreditation board and the number of methods or techniques being accredited, as well as travel and accommodation costs of the auditors during the assessment process. The composition of the auditors’ team and the length of the stay also depend on the complexity of the laboratory.

Some challenges experienced during the accreditation of the CISM laboratory included the scarcity of onsite specialised services for maintenance and calibration of laboratory equipment. Almost all reagents, materials, and equipment have to be imported, which may take several weeks to months to have them on site. The lack of local expertise is associated with increased costs to run the activities. The EQA is also challenging because foreign companies offer this service. This situation was critical in 2020 owing to the coronavirus disease 2019 (COVID-19) pandemic, the declaration of a state of emergency that affected the procurement of reagents with several delays leading to the observed lower client satisfaction in acquisitions and storage in 2020 (Figure 1).

We also noticed the lack of local reference ranges for haematology and biochemistry that reflect the local population in Mozambique.^26,27^ Most clinical laboratories in African countries use reference values provided by the equipment manufactures and/or described in the literature, which are based typically on data collected from European or North American populations. This gap represents an urgent need to establish local reference ranges, as many studies have shown that the references ranges from developed countries can differ from those obtained from populations living in Africa.^27,28,29,30^

Solutions to overcome some of these challenges include the service contract agreements with suppliers to guarantee the continuity of the laboratory services. The existence of back-up equipment and identification of referral laboratories, wherever possible, were also essential for the achievement of ISO 15189:2012 accreditation. Therefore, constant monitoring of quality indicators is essential to identify problems quickly and to follow up promptly. Indeed, the accreditation process does not end with the awarding of the certificate; rather, it is a continuous process with regular evaluations, usually annually, to guarantee that the laboratory always, at all times, maintains the quality system and in all areas.

### Conclusions

The accreditation of the CISM laboratory was a complex process, which was only possible with the involvement of the whole organisation. A continuous improvement process has been essential to achieve and maintain accreditation.
